# Skeletal muscle structural lipids improve during weight-maintenance after a very low calorie dietary intervention

**DOI:** 10.1186/1476-511X-8-34

**Published:** 2009-08-13

**Authors:** Steen B Haugaard, Allan Vaag, Huiling Mu, Sten Madsbad

**Affiliations:** 1 Dept. of Endocrinology, Copenhagen University Hospital, Hvidovre, Denmark; 2 Clinical Research Centre, Copenhagen University Hospital, Hvidovre, Denmark; 3 Dept. of Internal Medicine, Copenhagen University Hospital, Amager, Denmark; 4 Lund University, Lund, Sweden and Steno Diabetes Center, Gentofte, Copenhagen, Denmark; 5 Dept. of Pharmaceutical and Analytical Chemistry, Faculty of Pharmaceutical Sciences, University of Copenhagen, Denmark

## Abstract

**Background:**

The objective was to investigate in a group of obese subjects the course in skeletal muscle phospholipid (SMPL) fatty acids (FA) during a 24-weeks weight maintenance program, which was preceded by a successful very low calorie dietary intervention (VLCD). Special focus was addressed to SMPL omega-3 FA, which is a lipid entity that influences insulin action.

**Methods:**

Nine obese subjects (BMI = 35.7 ± 1.0 kg/m^2^), who had completed an 8 weeks VLCD (weight-loss = -9.7 ± 1.6 kg, P < 0.001), had obtained skeletal muscle biopsies (vastus lateralis) before and after a dietician-guided 24-weeks weight-maintenance program (-1.2 ± 1.5 kg, P = ns). SMPL FA composition was determined by gas liquid chromatography. During the preceding VLCD, insulin sensitivity (HOMA-IR) and glycemic control (HbA1c) improved but no change in SMPL omega-3 FA was observed. During the weight-maintenance program five subjects received the pancreas lipase inhibitor Orlistat 120 mg t.i.d. versus placebo.

**Results:**

HOMA-IR and HbA1c stabilized and SMPL total omega-3 FA, docosahexaenoic acid and ratio of n-3/n-6 polyunsaturated FA increased by 24% (P < 0.01), 35% (P < 0.02) and 26% (P < 0.01), respectively, whereas saturated and monounsaturated FA did not change. Plasma total-cholesterol and LDL-cholesterol, which decreased during the VLCD, reverted to pre-VLCD levels (P < 0.01). Orlistat therapy was associated with weight-loss (P < 0.05), trends for better glycemic control (P = 0.15) and greater increase in SMPL docosahexaenoic acid (P = 0.12) but similar reversal of plasma cholesterols compared to placebo.

**Conclusion:**

The data are consistent with the notion that greater SMPL omega-3 FA obtained during a weight-maintenance program may play a role for preserving insulin sensitivity and glycemic control being generated during a preceding VLCD.

## Background

Very low calorie dietary intervention (VLCD) has proven to be an efficient tool to provide weight-loss of 1-2 kg per week in obese subjects [1]. VLCD is often carried out for 4 - 12 weeks, and, if successful in achieving a weight loss, is likely to be associated with an improved lipid profile and insulin action [1-4]. It remains, however, a challenge to stabilize or further improve the results obtained during a VLCD both in terms of weight loss but also the metabolic parameters associated with a reduced risk of co-morbidity as cardiovascular disease and type 2 diabetes [1,4,5]. Intensified dietary advisory efforts after a VLCD may help in accomplishing these goals, but at best a stabilization of bodyweight may be achieved during this post-VLCD period [1,4,5].

We have recently reported that during 8 weeks VLCD, in which, as expected, plasma lipid profile and insulin sensitivity improved significantly after weight loss, the phospholipid fatty acid composition of the skeletal muscle cell membrane also improved in terms of desaturation [3]. As shown in several animal and human studies desaturation of skeletal muscle phospholipids is associated with improved insulin sensitivity [6-12]. However, improved insulin action may especially be associated with long-chain-polyunsaturated omega-3 fatty acids (LCPUFAn-3) in the structural lipids of skeletal muscle and among the LCPUFAn-3, docosahexaenoic acid (DHA) may be the most important fatty acid (FA) in this context [8,9,11,13,14]. We did not observe any net change in skeletal muscle phospholipid LCPUFAn-3 and especially DHA did not increase during the 8 weeks VLCD [3], which could be due to the longer retroconversion step of peroxisomal -oxidation in DHA formation [15] and rapid turnover of whatever docosahexaenoic acid was present.

The present study aimed to investigate the changes in skeletal muscle phospholipid FA composition during a period of weight stabilization following a successful VLCD in obese subjects. To this end, we examined the subjects in whom we previously reported desaturation in this lipid entity during 8 weeks VLCD [3]. These subjects were followed for an additional 24 weeks period of extensive dietary counseling and treatment with the pancreas lipase inhibitor, Orlistat versus placebo [5]. We report that the subjects during this period stabilized in weight and insulin action concomitant with increased LCPUFAn-3 especially the DHA in their skeletal muscle membrane, which coincided by a deterioration of their lipid profile to pre-VLCD levels. It is suggested that improvement in structural lipids during a weight stabilizing period following successful VLCD may help in preserving the improvement in insulin action obtained during the preceding VLCD.

## Methods

### Study subjects

Seventeen obese volunteers (13 women) were recruited from an ongoing dietary intervention study at our outpatient university-based diabetes and obesity clinic. Thirteen subjects (9 women) completed the 8 weeks VLCD and consented to continue on the present study and have obtained another muscle biopsy after 24 weeks on a dietary maintenance period. Data form the VLCD study has been published previously [3]. Selection criteria at baseline were abdominally obese patients (30 kg/m2 ≤ BMI < 40 kg/m2 and waist ≥ 92 cm (females) or waist ≥ 102 cm (males)), of age ≥ 18 years and < 65 years and with at least one of the following risk factors: a) early (i.e. only diet treated) type 2 diabetes mellitus (fasting plasma glucose ≥ 7 mM) or impaired fasting plasma glucose (6.1 mM ≤ FPG < 7 mM); b) dyslipidemia, HDL-C ≤ 0.9 mM (males) or HDL-C ≤ 1.1 mM (females) or serum triglycerides ≥ 2.3 mM but < 10 mM. Patients with a glycosylated hemoglobin (HbA1c) > 10% were excluded. Any ongoing medication for dyslipidemia and diabetes prohibited participation in the study, as did supplements of n-3 fatty acids (i.e. fish-oil), except from cod-oil in dietary doses. Informed written consent was obtained in accordance with Helsinki Declaration II. The local ethics committee approved the present substudy and the study was approved by the ethical committee of the cities of Copenhagen and Frederiksberg, Denmark (trial no. 01-363-98).

### Dietary intervention and study medication

Recommended energy level during the VLCD period was 600 - 800 Kcal/day, which was provided by the Nutrilett Intensive energy powder (Nycomed Pharma AS, Oslo, Norway) [16]. The Nutrilett formula provides 318 Kcal per 100 grams powder, i.e. proteins 36.8 grams, carbohydrates 30.7 grams and fatty acids 4.9 grams (1.3 gram saturated fatty acids, 1.9 gram linoleic (C18:2 n-6), and 0.3 gram linolenic (C18:3 n-3) fatty acids). During the 24 weeks "weight maintenance" period the subjects were prescribed a nutritionally balanced, hypocaloric diet designed to cause weight loss of 0.25 to 0.50 kg/week, with 30% of the calories as fat (optimally as 10% saturated, 10% monounsaturated and 10% polyunsaturated), 50% carbohydrate and 20% protein. At the beginning of the weight maintenance period subjects were also randomized (doubled blinded, ratio 1:1) to receive either the orally administered pancreas lipase inhibitor 120 mg t.i.d. or corresponding placebo [5]. The subjects consulted a registered dietician 6 times during the 8 weeks VLCD (i.e. at weeks 0, 1, 3, 5, 7 and 8) and 10 times during the 24 weeks weight maintenance period (i.e. weeks 1, 2, 3, 4, 6, 8, 12, 16, 20, and 24) for the purpose of weight control and guidance.

### Anthropometric measurements

Body weight and height were measured on a calibrated scale. Waist circumference was measured in the standing position between the top of the iliac crest and the lower rib margin on each side, while the patient exhaled, and with the tape parallel to the floor. Hip circumference was measured in the horizontal plane at the level of the maximal extension of the buttocks. Measurements of weight, height, waist, and hip were carried out in duplicate and mean values were noted. BMI was calculated as weight/height^2 ^(kg/m^2^). Total body fat mass and lean body mass were estimated by dual energy X-ray absorptiometry (DEXA) scanning (Norland Medical System XR-36, Fort Atkinson, WI, USA).

### Blood sampling and assays

Blood samples were collected after an overnight fast (≥ 8 hours), handled and analyzed by commercial available kits in accordance with the standard procedures for the central laboratory, Medi-Lab (Copenhagen, Denmark). The following blood variables were analyzed; HbA1c, plasma glucose, plasma insulin, plasma C-peptide, total cholesterol, HDL-cholesterol, LDL-cholesterol, and serum triglyceride.

### Muscle biopsy

A percutaneous muscle biopsy was obtained under local anesthesia using a Bergström needle (Depuy, AZ, USA) from the vastus lateralis muscle before and following the 8 weeks VLCD and after the 24 weeks weight maintenance period. The specimen was immediately and carefully dissected free of visible connective tissue, lipid and blood and frozen in liquid nitrogen and stored at -80°C until assayed.

### Skeletal muscle phospholipids and triglycerides

Extraction of skeletal muscle phospholipids and triglycerides in general followed the principle described by Folch et al [17]. Internal standards of C15:0 phosphatidylcholine and C15:0 triglycerides were added to samples of skeletal muscle tissue followed by extraction of the total lipid material with chloroform/methanol, 2:1, vol/vol, after homogenization with an Ultra Turrax homogenizer. The extracted lipids were separated into phospholipids and triglycerides by thinlayer chromatography (TLC) using a pre-manufactured silica plate (E. Merck, Germany). The kiesel gel bands containing the phospholipids and the triglycerides were scraped off the TLC plate and the lipids were extracted from the kiesel gel. The fatty acid profiles of phospholipids and triglycerides were determined by gas-liquid chromatography (GLC) of the fatty acid methyl esters using a Hewlett-Packard 6890 instrument (Hewlett-Packard, Germany) equipped with an SP2380 capillary column (60 m × 0.25 mm, ID, and film thickness 0.2 μm, Supelco, PA, USA) operated with temperature programming and using helium as carrier gas. Detection was by flameionisation. Fatty acids methylesters were identified by comparing their retention times with those of actual standards (Sigma Inc., MO, USA). The individual fatty acids were quantified and reported as their percentage of the total peak area. Only fatty acids constituting more than 0.1% of total peak area are reported. Content of triglycerides in each sample was calculated from the internal standard.

### Calculations

The homeostasis model assessment insulin resistance index (HOMA-IR) derives an estimate of whole body insulin sensitivity from fasting glucose and insulin concentrations [18]. Changes, when given in percentages, were calculated as the value after the intervention period minus the value before the intervention period divided by the latter value and multiplied with 100%.

### Statistics

All data are presented as mean ± SEM if not otherwise indicated. The paired student t-test was used to compare distribution of paired data sets. Statistical analyses were performed using SPSS version 12.0 (SPSS Inc., IL, USA). Statistical significance was accepted for P < 0.05.

## Results

Data are shown for the nine obese subjects (seven women) who had obtained skeletal muscle biopsy at the end of the preceding 8 weeks VLCD and after 24 weeks on a weight stabilization diet (Table 1). Thus four subjects out of the initial 13 subjects who had taken the biopsy after the VLCD period missed the final biopsy, which was due to unwillingness to undertake this procedure. There was no difference in baseline parameters among those who had taken a biopsy during the weight maintenance period and those who had not.

**Table 1:** Anthropometric and metabolic characteristics of study subjects after VLCD and during the weight maintenance period

	**After VLCD**		**During weight maintenance**				**Before VLCD**
	**Mean**	**SEM**	**Mean**	**SEM**	**% change**	**P**	**Mean**

Age (years)	52.4	3.3	52.8	3.3			52.2

Height (cm)	171.1	3.3					

BMI (kg/m^2^)	32.4	1.1	31.9	1.4	-1.3	ns	35.7*

Waist (cm)	104.3	1.5	103.3	2.5	-1.0	ns	111.3*

Hip (cm)	117.6	3.4	117.4	4.4	-0.1	ns	124.4*

Fat mass (kg)	38.5	3.8	35.8	4.1	-7.0	ns	44.8*

Lean mass (kg)	53.2	4.4	55.2	4.0	3.8	ns	56*

Weight (kg)	94.7	4.0	93.5	4.9	-1.3	ns	104.5*

							

Fp-glucose (mM)	6.2	0.2	6.5	0.2	6.0	ns	7.5*

Fp-insulin (pM)	57	6	69	9	20.4	ns	86*

Fp-C-peptide (pM)	841	87	878	88	4.4	ns	1084*

HbA1c (%)	6.1	0.2	6.1	0.2	0.3	ns	6.7*

							

HOMA-IR	2.2	0.3	2.8	0.5	28.9	ns	4.1*

Fp-cholesterol (mM)	5.2	0.4	6.4	0.4	22.1	<0.001	6.4*

Fp-LDL-cholesterol (mM)	3.3	0.4	4.0	0.3	19.0	<0.01	4.2*

Fp-HDL-cholesterol (mM)	1.2	0.1	1.4	0.1	10.8	<0.05	1.4*

Fs-triglyceride (mM)	1.4	0.1	2.2	0.4	55.8	ns	1.8

Data are from n = 9 subjects (7 females). For comparison data from before the VLCD are given for these subjects. Asterisks indicate P < 0.05 for comparison between "before" and "after" the preceding VLCD.

"% change" indicates the change relative to baseline level (i.e. after VLCD); BMI, body mass index; Fp, fasting plasma,; HbA1c, glycosylated hemoglobin; HOMA-IR, homeostasis model assessment; Fs, fasting serum

The subjects succeeded in stabilizing body weight, fasting plasma glucose, insulin, HbA1c, C-peptide and HOMA-IR during the weight maintenance period, whereas plasma lipids reverted to pre-VLCD levels (Table 1). Among the nine subjects who fulfilled this study, five subjects (four women) received the pancreas lipase inhibitor, Orlistat 120 mg t.i.d. The changes in bodyweight among the subjects on Orlistat compared to those on placebo showed that the former lost more weight (-3.9 kg vs. +2.2 kg, P < 0.05) and the Orlistat group also showed a trend for better glycemic control (HbA1c -0.1% vs. +0.2%, P = 0.15), but both sub-groups showed the same reversal of plasma cholesterols and plasma triglyceride towards pre-VLCD levels (data not shown).

**Figure 1:**
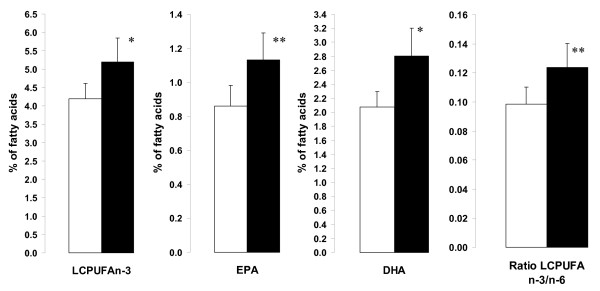
**The four diagrams show the key-changes in skeletal muscle phospholipid fatty acid composition during a period of weight stabilization following a VLCD**. Concentration of long-chain polyunsaturated fatty acids of the n-3 class (LCPUFAn-3) increased due to increased eicosapentaenoic acid (EPA, C20:5n-3) and docosahexaenoic acid (DHA, C22:6n-3); also the ratio of LCPUFA of the n-3 class versus the n-6 class increased. Data are Mean +/- SEM. White bars give data obtained at the end of the VLCD period (baseline) and black bars give data obtained 24 weeks later during the weight maintenance period. *, P < 0.05; **, P < 0.01.

Table 2 shows the changes in FA composition of skeletal muscle phospholipids during the 24 weeks weight maintenance period following the 8 weeks VLCD, pre-VLCD data are also given for comparison. Notable, the concentration of LCPUFAn-3 increased considerable due to increased DHA and EPA (eicosapentaenoic acid) and the ratio of LCPUFA n-3/n-6 increased (Figure 1). The precursor of LCPUFAn-3, α-linolenic acid (C18:3n-3) increased. No net changes in saturated or monounsaturated FA were observed during the 24 weeks period of weight maintenance. However, if we consider the pre-VLCD concentration of total saturated FA through to the weight maintenance period it decreased by 7.6%, P < 0.05. There was a trend for a greater effect on DHA in the Orlistat group compared to placebo (51% vs. 17%, P = 0.12) but no trends for differences in EPA (34% vs. 32%) or total LCPUFAn-3 (31% vs.14%).

**Table 2:** Skeletal muscle phospholipids of study subjects after VLCD and during the weight maintenance period

	**After VLCD**		**During weight maintenance**				**Before VLCD**
	**Mean**	**SEM**	**Mean**	**SEM**	**% change**	**P**	**Mean**

C14:0	0.41	0.02	0.53	0.04	28.8	<0.05	0.47

C16:0	16.63	0.83	16.23	0.45	-2.4	ns	17.93

C18:0	17.71	0.37	16.79	0.28	-5.2	ns	18.05

**Saturated**	35.01	0.69	33.96	0.36	-3.0	ns	36.73*

							

C16:1(n-7)	0.79	0.05	0.77	0.09	-2.5	ns	0.76

C18:1trans	0.53	0.09	0.75	0.11	41.8	ns	0.51

C18:1(n-9)	7.99	0.31	7.21	0.42	-9.7	ns	7.69

C18:1(n-7)	2.34	0.11	2.17	0.10	-7.2	<0.05	2.16*

**Monounsaturated**	11.88	0.40	11.19	0.59	-5.9	ns	11.27*

							

C18:2(n-6)	30.93	0.92	31.89	1.38	3.1	ns	31.75

C18:3(n-3)	0.31	0.05	0.46	0.04	48.0	<0.05	0.40

C20:3(n-9)	0.28	0.01	0.21	0.02	-23.2	ns	0.23

C20:3(n-6)	1.42	0.09	1.38	0.11	-2.9	ns	1.40

C20:4(n-6)	13.64	0.59	12.67	0.62	-7.1	ns	12.04*

C20:5(n-3)	0.86	0.12	1.13	0.16	31.6	<0.01	1.02*

C22:4(n-6)	0.54	0.09	0.48	0.03	-11.6	ns	0.42*

C22:5(n-3)	1.26	0.10	1.26	0.14	0.0	ns	1.08

C22:6(n-3)	2.08	0.22	2.81	0.40	35.1	<0.05	2.15

							

**PUFA**	51.13	0.80	52.27	0.97	2.2	ns	50.35
**LCPUFA**	20.31	0.67	20.24	1.11	-0.3	ns	18.54
**LCPUFAn-3**	4.20	0.41	5.20	0.65	23.8	<0.05	4.25
**LCPUFAn-6**	15.70	0.65	14.64	0.73	-6.7	ns	13.94*
**Ratio LCPUFA n-3/n-6**	0.098	0.012	0.124	0.017	25.8	<0.01	0.104

Data are from n = 9 subjects (7 females). For comparison data from before the VLCD are given for these subjects. Asterisks indicate P < 0.05 for comparison between "before" and "after" the preceding VLCD.

"% change" indicates the change relative to baseline level (i.e. after VLCD); FA, fatty acids; PUFA, polyunsaturated fatty acids; LCPUFA, long chain (i.e. ≥ C20) polyunsaturated fatty acids;

Table 3 shows the changes in intramyocellular triglyceride FA composition and total content of this depot lipid. Content of intramyocellular triglyceride decreased insignificantly by 2.3 mg/g wet tissue weight during the weight maintenance period. By including the VLCD period the total decrease in IMTG was 5.0 mg/g wet tissue weight (-24%, P = 0.15). During the 24 weeks weight maintenance period the general picture of changes in FA of intramyocellular lipid was a reduction in LCPUFAn-6 by 35%, P < 0.05 and LCPUFAn-3 by 64%, P < 0.01, whereas total monounsaturated FA showed a trend for increase of 2.0%, P = 0.07 and total saturated FA were unchanged. There were no trends towards a difference in effect on amount and composition of intramyocellular triglyceride between those who were treated with Orlistat compared to those on placebo (data not shown).

**Table 3:** Skeletal muscle triglycerides of study subjects after VLCD and during the weight maintenance period

	**After VLCD**		**During weight maintenance**				**Before VLCD**
	**Mean**	**SEM**	**Mean**	**SEM**	**% change**	**P**	**Mean**

C12:0	0.64	0.11	0.66	0.13	3.5	ns	0.62

C14:0	2.70	0.15	2.96	0.19	9.6	<0.05	2.80

C16:0	22.72	0.46	23.64	0.44	4.0	<0.05	23.56

C18:0	4.28	0.45	3.73	0.29	-12.8	ns	4.15

**Saturated**	30.55	0.97	31.21	0.86	2.2	ns	31.34

							

C14:1	0.41	0.04	0.47	0.05	16.1	<0.05	0.35

C16:1(n-9)	0.72	0.05	0.79	0.06	9.5	<0.05	1.02

C16:1(n-7)	5.56	0.53	6.52	0.48	17.3	<0.01	5.74

C18:1trans	1.19	0.08	1.23	0.13	3.0	ns	1.03

C18:1(n-9)	43.18	0.48	43.49	0.56	0.7	ns	42.73

C18:1(n-7)	2.63	0.12	2.56	0.11	-2.7	ns	2.98

C20:1	0.91	0.02	0.60	0.03	-34.7	<0.01	0.66

**Monounsaturated**	54.90	0.81	55.99	0.60	2.0	ns	54.72

							

C18:2(n-6)	10.86	0.32	10.40	0.34	-4.2	<0.05	10.81

C18:3(n-3)	1.04	0.10	1.00	0.10	-3.9	ns	1.11

C20:3(n-6)	0.26	0.02	0.19	0.03	-27.6	ns	0.21

C20:4(n-6)	0.49	0.04	0.40	0.06	-18.0	ns	0.36*

C22:3(n-3)	0.39	0.09	0.04	0.04	-89.8	<0.01	0.31

C22:5(n-3)	0.46	0.04	0.21	0.08	-54.7	<0.01	0.35*

C22:6(n-3)	0.55	0.12	0.31	0.14	-43.3	<0.05	0.46

							

**PUFA**	14.21	0.49	12.56	0.58	-11.6	<0.01	13.73

**LCPUFA**	2.45	0.24	1.37	0.29	-44.2	<0.01	1.86*

**LCPUFAn-3**	1.44	0.21	0.52	0.22	-64.3	<0.01	1.16*

**LCPUFAn-6**	1.00	0.07	0.65	0.11	-35.1	<0.05	0.70*

							

**Total IMTG **(mg/g wet tissue)	18.4	4.6	16.1	2.1	-12.5	ns	21.1

Data are from n = 9 subjects (7 females). For comparison data from before the VLCD are given for these subjects. Asterisks indicate P < 0.05 for comparison between "before" and "after" the preceding VLCD.

"% change" indicates the change relative to baseline level (i.e. after VLCD); FA, fatty acids; PUFA, polyunsaturated fatty acids; LCPUFA, long chain (i.e. ≥ C20) polyunsaturated fatty acids; IMTG, intramyocellular triglyceride

## Discussion

The major finding in the present pilot study on the fatty acid composition of structural lipids of skeletal muscle membrane in obese subjects during a weight stabilization period after a successful VLCD was that the important LCPUFAn-3 docosahexaenoic acid (DHA) and eicosapentaenoic acid (EPA) increased. This improvement obtained in FA composition of structural lipids during the weight stabilizing period after a VLCD may have provided assistance to preserve insulin action and glycemic control in face of reversal of plasma lipids to pre-VLCD levels. The data also provide some evidence to suggest that the positive effect of a pancreas lipase inhibitor as Orlistat on body weight in the post-VLCD period may be associated with a beneficial effect on the FA composition of skeletal muscle structural lipids.

VLCD has proven to be effective in the short run, whereas the post-VLCD period has been a period of massive failure to simply just maintain the achieved weight loss [1]. In this perspective close dietary advisory efforts may be of importance, but also anti-obesity medication may help in stabilizing body weight and may even facilitate further weight loss [1,5]. The pancreas lipase inhibitor Orlistat has proven an effective tool to achieve a surplus of weight loss compared to placebo in studies where all obese subjects were undergoing dietary guidance concomitant with anti-obesity medication [19]. Orlistat reduces the absorption of fat, including saturated fat from the intestinal with about 30%.

The present study aimed to obtain new knowledge about the changes in FA composition of phospholipids of skeletal muscle membrane during the setting of a post-VLCD period. Skeletal muscle is the major site of insulin action in terms of facilitating glucose metabolism. The FA composition of the skeletal muscle cell membrane is associated with insulin action such that a lower saturation and a higher concentration of LCPUFAn-3 facilitate insulin action [6,8-10,12,13,20-23]. The mechanism may involve changes in number of insulin receptors and facilitated insulin signal transduction in the skeletal muscle cell [7,14,24]. The phospholipid FA composition may be modified during moderate weight loss over 24 weeks as previously shown by us [9]. Thus, changes in LCPUFAn-3 especially the DHA of this lipid entity may confer improved insulin sensitivity [9]. As shown previously, the 8 weeks VLCD did not improve LCPUFAn-3 in skeletal muscle cell membrane [3]. This was speculated to be caused by a longer retroconversion step of peroxisomal -oxidation in DHA formation [15] or increased usage of the muscle cell membrane DHA.

The present study suggests that adherence to a diet over 24 weeks, which is able to stabilize a fast obtained weight loss during a VLCD, may further improve the FA composition of phospholipids in skeletal muscle of obese subjects. Whereas the preceding 8 weeks VLCD was associated with a reduction in saturated FA and increased monounsaturated FA contents, only changes in the LCPUFAn-6 class (increase) and not the LCPUFAn-3 class were obtained during this preceding VLCD. During the subsequent 24 weeks on a weight stabilizing diet, the contents of the class of LCPUFAn-3 increased, which was primarily due to the fact that both DHA and EPA increased during this period. DHA and EPA in cell membrane phospholipids have been associated with improved insulin action [11,13,25]. The ratio of n-3/n-6 LCPUFA also increased during this period, which may further indicate a more "healthy" muscle membrane in terms of a FA composition likely to facilitate insulin action. The magnitude of improvement of LCPUFAn-3, especially the increase in DHA and the ratio of LCPUFA n-3 vs. n-6 may translate into a relative improvement in insulin sensitivity of approximately 25% given the results from other studies as discussed previously [26]. Of note, no further improvement in saturated or monounsaturated FA was obtained during the 24 weeks of stable body weight after a VLCD, but throughout the total period of VLCD and weight maintenance the saturated FA of structural lipids decreased, which may also have aided in improving glucose metabolism [9]. The mechanisms behind the improved skeletal muscle phospholipid FA profile in this setting could be several. It may be that the preceding VLCD did not reveal an increased concentration of DHA due to the complex and longer duration of DHA formation [15]. A sustained dietary restriction program may increase level of LCPUFAn-3 as shown in the rat model [27]. Indeed, the changes in dietary FA composition during the weight maintenance period may have conferred the observed changes in the structural lipids FA composition of these obese subjects [8,9,11,28,29]. The results also support that the dietary source of LCPUFAn-3 had been indirect through providing increased amount of dietary linolenic acid, which could be elongated and desaturated in situ to EPA and DHA. Thus, the study provides an example of dissociation between effects of diet restriction on skeletal muscle structural lipid composition and weight loss per se in obese humans.

In accordance with the known effects of Orlistat on weight reduction and maintenance, we observed a significant difference in changes in weight during the 24 weeks post-VLCD between those treated with Orlitat versus placebo in our relatively small study population [5,19]. Accordingly, we cannot exclude the possibility that the trend towards a superior effect of Orlistat on the composition of the structural lipids to some extent may be due to a greater loss of body weight in the Orlistat arm. Nevertheless, this small pilot study obviously was not designed to address this aspect of Orlistat treatment, which needs to be addressed in a separate study.

A high amount of intramyocellular triglycerides in sedentary subjects has been associated with insulin resistance [30-34]. Taken together, the VLCD intervention followed by the 24 weeks weight maintenance period in the present study showed a trend for a 24% decline in intramyocellular triglycerides. Interestingly, the changes in the FA composition in intramyocellular triglyceride went opposite as compared to the changes in FA in phospholipids during the 24 weeks weight maintenance period. Thus the contents of LCPUFAn-3 and LCPUFAn-6 decreased in intramyocellular triglyceride. It should be acknowledged, however, that whereas the data to support an impact of the skeletal muscle FA composition on insulin action seems strong and consistent, the data to suggest an effect of the intramyocellular triglyceride FA composition playing some role for glucose metabolic parameters are scarce and contradicting [12,35-38]. Nevertheless, the obviously opposite net effect on the FA composition of intramyocellular triglyceride versus skeletal muscle phospholipids suggest different metabolism of these lipid entities, at least when evaluated during a dynamic setting after a VLCD.

## Conclusion

Considering the caveats of a small pilot study such as this, the present data suggest that following a successful 8 weeks VLCD, a 24 weeks period of weight maintenance program including the pancreas lipase inhibitor Orlistate may be associated with improvements in the FA composition of skeletal muscle phospholipids in terms of significant increases in EPA and DHA, which potentially may help in stabilizing insulin sensitivity in this setting.

## Abbreviations

VLCD: very-low-calorie dietary intervention; PUFA: polyunsaturated fatty acid; LCPUFA: long-chain PUFA; DHA: docosahexaenoic acid; EPA: eicosapentaenoic acid; IMTG: intramyocellular triglyceride; FA: fatty acid; HDL-C: high-density lipoprotein-cholesterol; HbA1c: glycosylated hemoglobin; LDL-C: low-density lipoprotein-cholesterol; HOMA-IR: homeostasis model assessment of insulin resistance.

## Competing interests

The authors declare that they have no competing interests.

## Authors' contributions

SBH conceived the study, participated in its design and coordination, performed muscle biopsies and drafted the manuscript. AV and SM participated in study design and coordination and helped to draft the manuscript. HM analyzed for IMTG and SMPL fatty acids and helped to draft the manuscript. All authors read and approved the final manuscript.
